# Influence of root canal taper on the measurement of two different electronic apex locators

**DOI:** 10.4317/jced.61352

**Published:** 2024-06-01

**Authors:** Francisco-Nathizael-Ribeiro Gonçalves, Amanda-Brito Santos, Ana-Letícia-Linhares-de Sousa Paula, Nathalia-Aguiar Freitas, Reuton-dos Santos-Palheta Filho, Fábio-Luiz-Cunha D’Assunção, Luciana-Maria-Arcanjo Frota, George-Táccio-de Miranda Candeiro

**Affiliations:** 1 DDS, MSc, Department of Dentistry, Unichristus, Fortaleza, Ceará, Brazil; 2 Undergraduate student in Dentistry School, Department of Dentistry, Unichristus, Fortaleza, Ceará, Brazil; 3 DDS, Department of Dentistry, Unichristus, Fortaleza, Ceará, Brazil; 4 DDS, MSc, PhD, Department of Dentistry, Federal University of Paraíba, João Pessoa, Brazil; 5 DDS, MSc, PhD, Department of Dentistry, Uninta, Sobral, Ceará, Brazil; 6 DDS, MSc, PhD, Department of Dentistry, Unichristus, Fortaleza, Ceará, Brazil

## Abstract

**Background:**

This research aimed to analyze the influence of root canal taper on the accuracy of two Electronic Apex Locators (EALs).

**Material and Methods:**

Twenty-five disto-vestibular roots from extracted human upper molars belonging to the tooth bank were used in this study. To determine the File Position (FP), access was made using a spherical diamond tip #1014, and the crowns were sectioned using a diamond tip #3080. The initial anatomic file used was a size K #10, which was introduced into the root canal until its tip was visualized (foraminal patency) with the aid of a clinical microscope (16X magnification). Teeth without foraminal patency and calcifications were excluded from the study. Odontometric readings were performed using two different EALs (Root ZX II and Romiapex A-15), considering the electronic reference point 0.0 (apex) for each device. All measurements were taken in triplicate, and the arithmetic mean of the three values was used. Digital calipers were used to record the measurements, which were then entered into an Excel spreadsheet. After visual verification using file K #10, the canals were instrumented with a #25.01 file to standardize the apical region, then successively instrumented with files #25.02, #25.04, #25.06, #25.08, #25.10, and #25.12, with electronic odontometry checked after each instrumentation using #25.02. Measurement 0.0 was adopted, with error margins of ±0.5 and ±1.0. Discrepancies between visual and electronic readings were statistically analyzed using ANOVA and Bonferroni tests, with significance considered when *P*<0.05.

**Results:**

Using the 0.0 mark and a ±1.0 error margin, it was observed that readings from the devices were similar in canals with different tapers (*P*>0.05), showing a tendency towards underestimation. However, when using the measurement variation margin of ±0.50, a statistically significant difference was found in the Romiapex A-15 group (*P*=0.0248) when comparing the results of the two EALs.

**Conclusions:**

Therefore, it was concluded that the canal taper did not significantly influence the accuracy of the evaluated EALs, using the reference point 0.0. When using the ±0.5 variation margin, the Romiapex A-15 device showed greater accuracy, and finally, at the ±1.0 error margin, both EALs exhibited excellent precision.

** Key words:**Endodontics, Odontometry, Eletronic Apex Locator, Root Canal Preparation.

## Introduction

There is a correlation between the success of endodontic treatment and the modeling and disinfection of the entire root canal system, followed by a hermetic obturation. Therefore, the professional must accurately determine the apical constriction (AC) to carry out subsequent treatment steps (1).

The precise establishment and maintenance of the working length (WL) are of fundamental importance for adequate endodontic treatment (2,3). Anatomical studies show that the AC is located between 0.5 and 1.0 millimeters (mm) from the major foramen³. A measurement shorter than the WL leads to inadequate root canal cleaning, while a measurement beyond the WL results in damage to the periapical tissue, which may delay or prevent healing (4,5).

In the literature, there are three methods to determine the endodontic WL: tactile, radiographic, and electronic methods (6). The use of cone-beam computed tomography (CBCT) is also mentioned as an effective method for obtaining WL during endodontic treatment (7). However, the radiographic method, although widely used, is susceptible to failures such as image distortions, superimposition of anatomical structures, and the inability to identify the precise location of the cemento-dentinal junction (6).

The use of electronic apex locators (EALs) for assessing canal length has gained popularity and eliminated problems associated with measurement through radiographs or tactile sensitivity (8). Depending on the direction and extent of root curvature and the position of the apical foramen in association with the anatomical apex, radiographic WL measurement can be extremely inaccurate (9). Literature describes several factors that can interfere with the accuracy and stability of odontometric measurements using EALs, such as the quality of isolation, presence of metal restorations, moisture in the pulp chamber, presence of perforations, anatomical variations, communicating root resorptions, and incomplete root formation (10). The choice of a file compatible with the apical foramen is important to establish the proper file positioning and minimize the risk of instrument displacement. An additional factor that may interfere with instrument compatibility with the apical foramen is pre-enlargement of the canal (11).

At present, no research has evaluated whether the taper of root canals post-instrumentation interferes with the determination of root canal lengths. Thus, the aim of this study was to examine the influence of root canal taper on the readings of electronic apex locators (Root ZX II and Romiapex A15). The null hypothesis considered was that the electronic apex locators would not be influenced by the taper of the root canal.

## Material and Methods

Initially, the present research was approved by the Research Ethics Committee of Centro Universitário Christus (UNICHRISTUS) under protocol number 4.730.488/2021.

-Sample Selection

The experiment’s methodology was based on previous studies (Lima *et al*. 2023). Twenty-five disto-vestibular (DV) canals from fully formed, straight upper molar roots were used. These were donated by patients through the Free and Informed Consent Form (FICF) to the tooth bank of Centro Universitário Christus.

Severely calcified canals or those impossible to achieve foraminal patency were excluded from the research, as well as roots where there was difficulty in measuring the length using the EALs. Teeth with cracks and/or fractures and roots showing dilaceration were also excluded.

The selected dental elements were stored refrigerated in a 0.2% thymol solution until the root canal instrumentation procedures were carried out. The study commenced only after approval by the Human Research Ethics Committee.

-Sample Preparation

**Figure 1:**
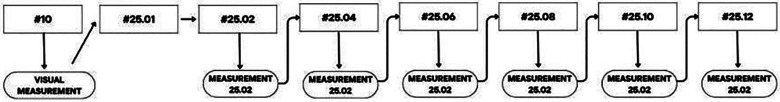
Flowchart of root canal preparation, visual and electronic measurements using EALs.

The previously selected teeth were radiographed in the buccal-palatal and mesio-distal directions to classify them within the selection criteria. The radiographs were analyzed using Image J software to confirm that the apical region had a diameter less than 250 micrometers. Subsequently, the coronal opening was performed using a 1014 HL diamond tip (KG Sorensen, São Paulo, Brazil) at high rotation, finishing with a 3080 diamond tip (KG Sorensen, São Paulo, Brazil). Following this, with the aid of a digital caliper (Mitutoyo, Suzano, Brazil) and a 3080 diamond tip (KG Sorensen, São Paulo, Brazil), coupled to a high-speed handpiece under abundant irrigation, all teeth had their occlusal surface worn down to flatten the face and standardize the tooth length to 15 mm (Fig. 1). This was done because the length of one of the instruments used in the experiment, the #25.12 file (SybroEndo, Glendora, United States), was 17 mm. This procedure also aimed to facilitate the positioning of the silicone stopper during the WL measurement, enhancing the precision in measuring.

-Verification of Odontometry by Direct (Visual) Method

The #10 file (Dentsply Maillefer, Ballaigues, Switzerland) was inserted into the AF until its tip was visualized through it with the assistance of a clinical microscope at 16x magnification (Alliance, São Carlos, SP, Brazil), precisely overlapping the AF. At this point, the silicone stopper placed on the file was stabilized on the tooth’s occlusal surface. The instrument was then removed from the canal, and its measurement was taken using a digital caliper (0.001 mm) (Mitutoyo, Suzano, SP, Brazil) to determine the CRC. Measurements were taken in triplicate, and from the obtained values, an average was calculated and considered as the initial length.

-Verification of Odontometry by Electronic Method of Apex Location

Following the odontometric assessment by the visual method, all specimens were instrumented with rotary file #25.01 (Easy, Belo Horizonte, Brazil) to standardize the apical region of the sample at 250 micrometers. Finally, the specimens were instrumented throughout the root canal extension with files of different tapers (#25.02, #25.04, #25.06, #25.08, #25.10, and #25.12). These files were used progressively, and subsequent odontometric measurements were performed using the Electronic Apex Locators (EALs): Root ZX II (J Morita, Tokyo, Japan) and Romiapex A-15 (Romidan, Kiryat-Ono, Israel) coupled with a #25.02 file (Fig. 1).

The chemomechanical preparation was performed using the VDW Silver motor (VDW GmbH) at 350 RPM and 300 g.cm, irrigated with 2.5% sodium hypochlorite solution (Rioquimica, SP, Brazil) using a luer lock irrigation syringe (BD Desrcarpack, SP, Brazil) and NaviTip irrigation cannula (Ultradent, Indaiatuba, São Paulo). Subsequently, excess solution in the pulp chambers was removed using an EndoFlex endodontic aspirator (Maquira, Maringá, Paraná).

Next, prior to the odontometric measurements and to simulate the oral environment, the specimens were mounted in an acrylic device and embedded in a conductive gel medium (Carbopol Gel, NaCl 0.9% and KCl 2% - FarmaVie Pharmacy, Fortaleza, Brazil). For this analysis, Root ZX II and Romiapex A-15 were used.

The electronic reference point was considered 0.0 (apex) on each device. Electronic measurements were performed according to the presented scheme below. Each measurement was taken in triplicate, and the obtained data were compared to the reference measurement.

For each measurement, the file was slowly inserted into the root canal until the display showed the apex marking (0.0) along with the audible signal. The silicone stopper was adjusted in the coronal portion. The measurement was considered correct if the instrument remained stable at the length for up to 5 seconds. Then, the file was removed from the canal, and the distance between the silicone stopper and the file tip was measured using a digital caliper. For a more accurate measurement within an accepted range, a 3.5x magnifying loupe (Bio-Art, São Paulo, Brazil) was used. Only one calibrated operator performed the laboratory steps. Each measurement was performed in triplicate, and the mean values were subsequently analyzed. Odontometric readings were taken with the #25.02 file as the canal was progressively instrumented with files of different tapers, according to the flowchart below.

.Data Collection and Statistical Analysis

After the measurements were tabulated in Microsoft Excel® spreadsheets, the quantitative data were subjected to the Kolmogorov-Smirnov normality test, expressed as mean and standard deviation, and analyzed using ANOVA followed by the Bonferroni post-test. Subsequently, the measurements were categorized based on the clinical relevance of tooth unit size measurement between ±0.50 and ±1.00. The data were expressed as absolute frequency and percentage and analyzed using the chi-square test. The significance level of the study was set at 5%.

## Results

**Figure 2:**
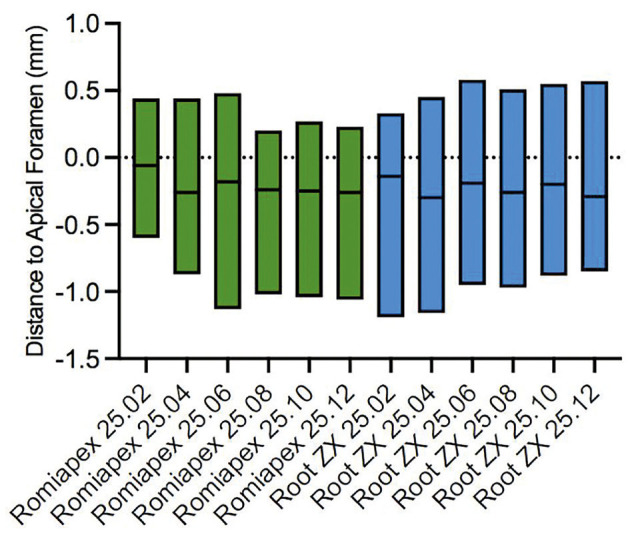
Average and Standard Deviation (SD) of measurements performed by EALs using 25.02 files in root canals shaped with different tapers.

Figure 2 presents the mean and standard deviation after instrumentation with files of different tapers (#25.02, #25.04, #25.06, #25.08, #25.10, and #25.12) and readings with instrument #25.02. It is observed that all means show underestimations ( Table 1, Fig. 2); however, there was no statistically significant difference when using the Root ZX II and Romiapex A-15 devices (*P*=0.3799).

In  Table 2, a predominance of underestimations can be seen when readings are taken with the Romiapex A-15 device, with most measurements falling within the range of apex distance from -0.50 to -0.01. It can also be noted that there are no measurements marking beyond the apex in the range ≥ 0.51.

In  Table 3, there is again a predominance of underestimations when readings are taken with the Root ZX II device, and also that most measurements fall within the range of apex distance from -0.50 to -0.01, as observed when using the Romiapex A-15 device.

When the results within the same group are compared, there is a statistically significant difference with the use of Romiapex A15, using the ±0.50 mm variation margin, demonstrating that this locator’s use is influenced by the file fit to the canal walls during foraminal localization, meaning that the measurements were more accurate in less widened canals. However, when comparing within the Root ZX II group, there is no significant difference within the ±0.50 mm variation margin, indicating that the accuracy of the second EAL is less influenced by the canal taper. Comparing both groups of EALs also shows a statistically significant difference, highlighting greater precision of the Romiapex A15, within the ±0.50 mm error margin. There is no difference within the same groups and between the groups in the range varying from ±1.00 mm ( Table 4).

## Discussion

The localization of the apical foramen (AF), and consequently the working length (WL), is the initial step in determining the boundaries for biomechanical preparation and root canal filling. The success of endodontic treatment is closely linked to maintaining the root canal filling limited to the region near the apical constriction (AC), approximately 0.5 mm short of the apical foramen (1-3,12,13).

Therefore, the use of Electronic Apex Locators (EALs) as a tool to determine the working length is strongly supported by the literature (14-16). Siqueira (17) stated it was impossible to precisely locate the working length through radiographic methods. However, to date, few studies have related the taper and adjustment of the endodontic instrument to the internal walls of the root canal to the accuracy of EALs.

While it is more common for scientific studies to assess the accuracy of EALs by comparing them with visual odontometry methods, recent studies, such as Suguro *et al*. (15) and De-Deus *et al*. (18), utilize micro-CT images to evaluate locator accuracy. Currently, micro-CT imaging is considered the most precise method to assess the internal structure of the root canal system. Additionally, micro-CT images are non-destructive research tools that can effectively identify, assess, and measure the root canal system in three dimensions.

The experimental model used in this study was similar to those used in previous works. For its execution, biomechanical preparation and measurements were carried out on the roots of upper molars, teeth widely subjected to endodontic treatments and previously used in prior studies (16).

The distal-buccal root of upper molars was employed in the experiment due to standardization reasons for the specimens. These roots tend to have straighter canals and a reduced diameter, making them more easily adaptable to the taper (configuration) of the files used in instrumentation. Furthermore, the cross-sectional shape of distal-buccal canals is more circular, reducing bias found in studies using root canals with isthmuses.

Studies conducted by Ibarrola *et al*. (19), Pécora *et al*. (20), Camargo *et al*. (20), and Carpena *et al*. (22) concluded that the endodontic pre-enlargement maneuver in the cervical and middle regions influenced the determination of the AP position. This maneuver enabled the file to consistently reach the AP, thereby increasing the effectiveness of the LEF.

In this study, three precision variables were adopted: 0.0, ± 0.50 mm, and ± 1.0. The first variable is the most precise, as the device indicates the AP position. The second variable has a tolerable margin of error but can be challenging to reproduce clinically since millimeter rulers lack 0.5 mm markings. The last variable represents a clinically achievable margin of error.

The most widely used electronic apex locator (EAL) in academic and clinical settings is the Root ZX, which demonstrates high accuracy rates. Vasconcelos *et al*. (23) and Plotino *et al*. (24) reported accuracy rates exceeding 90%, making it a pioneering tool in this field.

Aguiar *et al*. (25) also emphasized the accuracy of the Root ZX device. Consistent with previous findings, the present study concluded that there is no influence of the adjustment of the endodontic instrument to the canal walls on the accuracy of foraminal localization when using the Root ZX device. However, Ebrahim *et al*. (26) claimed that the file size interferes with the determination of the foraminal position, but both the ROOT ZX and the Foramatron D10 proved more reliable in determining the CT of teeth with an enlarged FA when using a snugly fitted file. In the current study, there was no statistical difference within the same group studied, nor between the groups, where all readings were statistically similar. However, it was observed that by adopting the distance variation of ± 0.50 mm from the FA, the Romiapex A-15 EAL showed interference in accuracy when the canal taper increased, demonstrating more precision when the instrument fit snugly into the canal during odontometry.

De-Deus *et al*. (18) stated that the precision of the ROOT ZX II showed an accuracy of 100% (within the tolerance margin of ±0.50 mm), yet the present study found a percentage ranging from 84% to 90.7%.

Vasconcelos *et al*. (23) found that the Novapex precision provided accepTable measurements regardless of instrument adjustments. However, there are divergent results in the present study when analyzing Root ZX data, which showed improved performance when a snugly fitted file was used.

Soares *et al*. (5) stated that the choice of a file compatible with the CA is important to establish the appropriate positioning of the file in locating the CRC, preventing the file from losing stability. Herrera *et al*. (27) concluded that the accuracy of the Root ZX EAL varied depending on the CA diameter, stating that the device’s accuracy gradually decreased as the foramen widened. Therefore, the results of the current study can be justified, as the root canals, before electronic measurements, had their apical region standardized to 250 micrometers using a #25.01 file, and all subsequent instruments had the same diameter at their active tip (250 micrometers), including the file used for measurement (#25.02). This demonstrates a possible greater relationship of EAL accuracy with the adjustment of the file to the foraminal region rather than with the entire canal length, as there was no statistically significant difference when analyzing the groups individually or when comparing them, adopting the range of 0.0 and ±1.0 mm.

Recently, Paiva *et al*. (28) observed that thermal treatment and the diameter of the apical preparation did not influence the accuracy of determining the root canal length.

Previous studies report that submeasurements are more related to measurements using smaller diameter instruments and those with less adaptation to the canal (26,14). In the present study, it was observed that all averages showed submeasurements, which may have been due to the lack of adaptation to the canal structure, as the instruments were more adapted to the diameter of the established apical foramen during instrumentation.

## Conclusions

Thus, it was concluded that the taper of the root canals were no significantly influence the accuracy of the evaluated electronic foraminal locators.

## Figures and Tables

**Table 1:** Averages, Standard Deviation (SD), maximum and minimum values of measurements by EALs performed with 25.02 files in root canals shaped with different tapers.

	ROMIAPEX A15						ROOT ZX II					
	25.02	25.04	25.06	25.08	25.10	25.12	25.02	25.04	25.06	25.08	25.10	25.12
Average	-0,06	-0,25	-0,16	-0,30	-0,30	-0,31	-0,21	-0,30	-0,18	-0,21	-0,22	-0,25
SD	0,31	0,35	0,38	0,32	0,34	0,32	0,39	0,39	0,36	0,34	0,35	0,35
Maximum	0,44	0,44	0,48	0,20	0,27	0,23	0,33	0,45	0,58	0,51	0,55	0,57
Minimum	-0,60	-0,87	-1,13	-1,02	-1,04	-1,06	-1,19	-1,16	-0,95	-0,97	-0,88	-0,85

P=0,3799 (ANOVA test)

**Table 2:** Absolute and percentual analysis of measurements in relation with apical foramen in root canals shaped with different tapers using RomiApex A15 device.

ROMIAPEX A15												
	25.02		25.04		25.06		25.08		25.10		25.12	
	n	%	n	%	n	%	n	%	n	%	n	%
> (-1.00)	0	0.0	0	0.0	2	2.7	1	1.3	2	2.7	1	1.3
(-1.00) a (-0.51)	4	5.3	7	9.3	5	6.7	7	9.3	8	10.7	9	12.0
(-0.50) a (-0.01)	39	51.3	56	74.7	41	54.7	55	73.3	53	70.7	50	66.7
0.00	0	0.0	0	0.0	0	0.0	0	0.0	0	0.0	3	4.0
0.01 a 0.50	33	43.4	12	16.0	27	36.0	12	16.0	12	16.0	12	16.0
0.51 - 1.00	0	0.0	0	0.0	0	0.0	0	0.0	0	0.0	0	0.0
> (1.00)	0	0.0	0	0.0	0	0.0	0	0.0	0	0.0	0	0.0
Total	75	100.0	75	100.0	75	100.0	75	100.0	75	100.0	75	100.0

**Table 3:** Absolute and percentual analysis of measurements in relation with apical foramen in root canals shaped with different tapers using Root ZX II device.

ROOT ZX II												
	25.02		25.04		25.06		25.08		25.10		25.12	
	n	%	n	%	n	%	n	%	n	%	n	%
> (-1.00)	2	2.7	3	4.0	0	0.0	0	0.0	0	0.0	0	0.0
(-1.00) a (-0.51)	8	10.7	9	12.0	7	9.3	8	10.7	9	12.0	10	13.3
(-0.50) a (-0.01)	41	54.7	45	60.0	47	62.7	43	57.3	44	58.7	44	58.7
0.00	0	0.0	3	4.0	2	2.7	0	0.0	0	0.0	0	0.0
0.01 a 0.50	24	32.0	15	20.0	19	25.3	22	29.3	20	26.7	19	25.3
0.51 - 1.00	0	0.0	0	0.0	0	0.0	2	2.7	2	2.7	2	2.7
> (1.00)	0	0.0	0	0.0	0	0.0	0	0.0	0	0.0	0	0.0
Total	75	100.0	75	100.0	75	100.0	75	100.0	75	100.0	75	100.0

**Table 4:** Percentual (%) of acuracy of each EALs, considering variations of tolerance (±0.50mm and ±1.00mm) in measurements performed with each device using 25.02 file.

	Between –0.50 and +0.50 mm			Between –1.00 and +1.00 mm		
Final Shaping File	Romiapex A15	Root ZX	p-value^*^	Romiapex A15	Root ZX	p-value^*^
25.02	94.7	86.7		100.0	97.3
25.04	90.7	84.0		100.0	96.0
25.06	90.7	90.7		97.3	100.0
25.08	89.3	86.7		98.7	100.0
25.10	86.7	85.3		97.3	100.0
28.12	86.7	84.0		98.7	100.0
Average	89.8^a^	86.2^b^	0.024	98.7^a^	98.9^a^	0.402

## Data Availability

The datasets used and/or analyzed during the current study are available from the corresponding author.
